# Functional and structural insights into cyanobacterial CO_2_ concentrating mechanisms: from compartmentalization to regulation

**DOI:** 10.1111/tpj.70974

**Published:** 2026-06-09

**Authors:** Erik Zimmer, Carolin Poppitz, Stephan Klähn, Khaled A. Selim

**Affiliations:** ^1^ Organismic Interactions Department Interfaculty Institute of Microbiology and Infection Medicine, Tübingen University 72076 Tübingen Germany; ^2^ Department of Solar Materials Biotechnology Helmholtz Centre for Environmental Research—UFZ Permoserstraße 15 04318 Leipzig Germany; ^3^ Microbial Biochemistry Group Institute of Phototrophic Microbiology, CEPLAS cluster of Excellence, Heinrich‐Heine University Düsseldorf Universitätsstraße 1 Düsseldorf 40225 Germany

**Keywords:** bacterial microcompartment, bicarbonate transporters, carbonic anhydrase, carboxysomes, CCM, CO_2_ metabolism, cyanobacteria, engineering CO_2_ fixation, photosynthesis and CO_2_ fixation, RuBisCO enzyme

## Abstract

Cyanobacteria are photoautotrophic microorganisms that fix CO_2_ through oxygenic photosynthesis during the day and rely on heterotrophic metabolism at night. In nature, the availability of inorganic carbon (Ci) is often limited, posing a major constraint on photosynthetic efficiency. To overcome this, cyanobacteria have evolved a sophisticated CO_2_‐concentrating mechanism (CCM) that enhances the catalytic performance of the primary carboxylating enzyme, ribulose‐1,5‐bisphosphate carboxylase/oxygenase (RubisCO). The CCM functions by elevating intracellular CO_2_ concentrations around RubisCO to suppress its oxygenase activity and enhance CO_2_ fixation efficiency. Central to this system is the carboxysome, a proteinaceous microcompartment that encapsulates RubisCO and carbonic anhydrase, facilitating efficient conversion of bicarbonate (HCO_3_^−^) to CO_2_ and its subsequent fixation. This is complemented by multiple Ci transporters that mediate active uptake of CO_2_ and HCO_3_^−^. Five major transport systems have been characterized: two specialized NDH‐1 complexes for CO_2_ transport and its conversion into HCO_3_^−^, and SbtA, BicA, and BCT1 for HCO_3_^−^ uptake. Recent structural studies on CCM uptake systems have revealed key mechanisms of HCO_3_^−^ transport, CO_2_ hydration and transport coupling. These insights provided a deeper understanding of how these systems enhance Ci acquisition and maintain photosynthetic efficiency across diverse environmental conditions and various CO_2_ regimes. Moreover, the CCM is tightly regulated at both transcriptional and post‐translational levels to balance energy usage and carbon demand. This review outlines our current insights into the molecular architecture, transport dynamics, and regulatory networks of the cyanobacterial CCM, emphasizing its critical role in photosynthesis and its potential as a model for bioengineering enhanced CO_2_ fixation or for engineering synthetic bacterial microcompartments.

## Significance Statement

Life on Earth depends on photosynthetic CO_2_ fixation via the Calvin–Benson–Bassham cycle to form organic carbon. This process evolved first in cyanobacteria and was later conveyed to eukaryotes, giving rise to plastids in algae and plants. To cope with low atmospheric CO_2_ concentrations that developed over the course of evolution, cyanobacteria evolved CO_2_‐concentrating mechanisms (CCMs), which elevate CO_2_ levels in the vicinity of RubisCO, the main CO_2_ fixation enzyme.

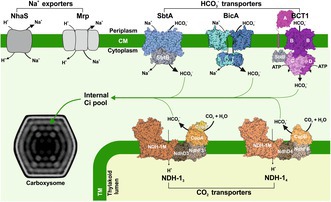

## THE CARBOXYLATION REACTION OF RubisCO LIMITS PHOTOSYNTHETIC EFFICIENCY

While CO_2_ is the major anthropogenic greenhouse gas driving climate change, it also represents the primary inorganic carbon (Ci) source for autotrophic organisms. These organisms are typically found at the basis of the food chain, because primary production via biological CO_2_ fixation is the fundament of building organic matter and biomass at global scale. Plants, eukaryotic algae, and cyanobacteria represent the special group of autotrophs that perform oxygenic photosynthesis. They convert CO_2_ into organic molecules and biomass, relying on only light energy and water as an electron donor, while releasing oxygen (O_2_) as a byproduct. Ribulose‐1,5‐bisphosphate carboxylase/oxygenase (RubisCO) serves as the key enzyme for Ci assimilation in these organisms. It catalyzes the carboxylation of ribulose‐1,5‐bisphosphate (RuBP) with CO_2_, producing two molecules of 3‐phosphoglycerate (3‐PGA). This reaction represents the rate‐limiting step of the Calvin–Benson–Bassham (CBB) cycle, often referred to as the ‘dark reactions’ (Barker et al., 1956). In this cycle, the energy‐rich cofactors ATP and NADPH, produced by photosynthetic light reactions, drive the formation of 3‐phosphoglycerate (3‐PGA) and further organic compounds to concurrently regenerate RuBP, the substrate for RubisCO. A recent structural study of RubisCO in complex with the transition‐state analog 2‐carboxyarabinitol‐1,5‐bisphosphate revealed protonation equilibria within its active site. Surprisingly, it also showed structural flexibility across large protein regions despite the tight ligand binding (Croy et al., 2025).

In addition to facilitating RuBP carboxylation, RubisCO can also catalyze its oxygenation, using O_2_ instead of CO_2_ as a substrate, generating one molecule each of 3‐PGA and 2‐phosphoglycolate (2‐PG) (Ogren, 2003). As 2‐PG cannot re‐enter the CBB cycle, its carbon is effectively lost from net CO_2_ assimilation. Moreover, 2‐PG is toxic to the cell, as it inhibits key CBB enzymes triose‐phosphate isomerase and sedoheptulose‐1,7‐bisphosphatase and must therefore be salvaged in the energetically costly multistep photorespiratory pathway (Flügel et al., 2017; Hagemann et al., 2016). However, this inhibitory effect has so far only been demonstrated in plants and has not yet been directly investigated in cyanobacteria, although a similar effect is expected. RubisCO's detrimental oxygenase activity arises from its dual substrate specificity for CO_2_ and O_2_, a relic of its evolutionary origin under an ancient atmosphere with a vastly different gas composition (Whitney et al., 2011). Geological evidence indicates that oxygenic photosynthesis might have emerged as early as 3.8 billion years ago (Ga), at a time when the atmosphere was essentially anoxic but contained high concentrations of CO_2_ (Buick, 2008; Lehmer et al., 2020). The evolution and spread of oxygenic photosynthetic organisms ultimately lead to the Great Oxidation Event ~2.3–2.4 Ga, during which atmospheric O_2_ levels rose dramatically over a geologically short interval (Buick, 2008). As a consequence of this long‐term biogeochemical transformation, present‐day atmospheric compositions stabilize at approximately 21% O_2_ and ~400 ppm (0.04%) CO_2_. These new conditions increased the frequency of RubisCO's oxygenation reactions, exerting strong selective pressure on photoautotrophs to either adapt the enzyme itself or develop CO_2_‐concentrating mechanisms (CCMs) that improve intracellular conditions for the carboxylation reaction, particularly in cyanobacteria and algae. In cyanobacteria, consistent with the co‐evolution of RubisCO alongside effective CCMs, a common signature of high catalytic turnover of RubisCO was found coupled with low CO_2_ affinity, despite significant strain‐specific differences in RubisCO's kinetics and CCM performance (Aguiló‐Nicolau et al., 2026).

Evolutionarily, RubisCO enzymes have evolved a higher affinity for CO_2_ and improved CO_2_/O_2_ selectivity by acquiring an accessory subunit that became essential before the rise of atmospheric oxygen, albeit at the cost of reduced catalytic turnover rates (Galmés et al., 2014; Schulz et al., 2022). To compensate for this reduced catalytic efficiency, C_3_ land plants increased RubisCO abundance, making it the most abundant enzyme on earth (Bar‐On & Milo, 2019; Raven, 2013). Despite these adaptations, RuBP oxygenation in C_3_ plants still accounts for approximately 30% of RubisCO's reactions, while simultaneously up to 25% of total cellular nitrogen is invested in protein synthesis of RubisCO in photosynthetically active cells (Luo et al., 2021). Plant species with a specialized C_4_ metabolism, such as maize or sugarcane, minimize photorespiration by spatially separating the initial CO_2_ fixation from the CBB cycle. Thereby O_2_ is effectively excluded from the bundle sheath cells containing RubisCO while the CO_2_ concentrations are elevated around the enzyme (Majeran et al., 2008; Sage et al., 2012). Similarly, many algal species employ pyrenoids, specialized subcellular microcompartments of different algal species, that concentrate CO_2_ to saturate RubisCO and enhance carboxylation efficiency (Rae et al., 2013). The relatively low specific activity and tendency to accept O_2_ as a substrate have made RubisCO an attractive yet challenging target for enzyme engineering. Some efforts have led to the identification of thermostable RubisCO variants, which convey resilience to otherwise detrimental amino acid exchanges (Hoffmann, Schuppe, et al., 2025). Furthermore, high‐fitness variants showed reduced catalytic efficiency for oxygenation and, in one instance, increased carboxylation turnover (Hoffmann, Knave, et al., 2025).

## THE CYANOBACTERIAL CCM COMBINES Ci UPTAKE SYSTEMS AND A PROTEINACEOUS MICROCOMPARTMENT

**Figure 1:**
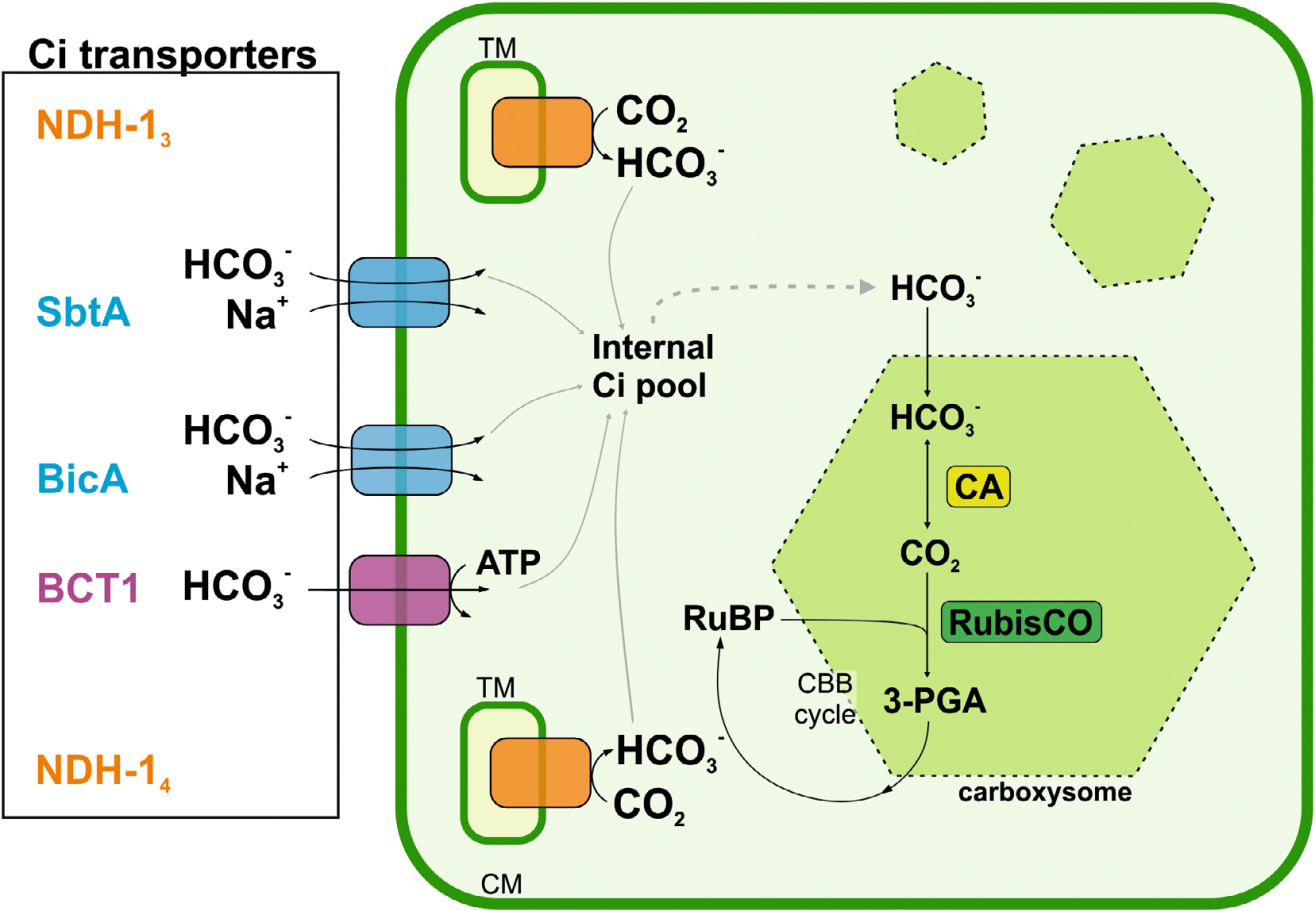
Schematic overview of the cyanobacterial carbon‐concentrating mechanism. Cyanobacteria possess five distinct CO_2_ and bicarbonate (HCO_3_^−^) uptake systems that collectively elevate intracellular inorganic carbon (Ci) levels. Specialized NDH‐1 complexes NDH‐1_3_ and NDH‐1_4_ (orange) convert CO_2_ to HCO_3_^−^, while sodium‐dependent bicarbonate transporters SbtA and BicA (blue), and the ABC transporter BCT1 (purple) translocate HCO_3_^−^. Accumulated HCO_3_^−^ diffuses into carboxysomes, where carbonic anhydrases (CA) catalyze its interconversion to CO_2_. The coordinated action of active Ci import, microcompartmentalization, and enzymatic generation of saturating CO_2_ concentrations around ribulose‐1,5‐bisphosphate carboxylase/oxygenase (RubisCO) minimizes its oxygenase activity and enables efficient carboxylation. 3‐PGA, 3‐phosphoglycerate; CM, cytoplasmic membrane; RuBP, ribulose‐1,5‐bisphosphate; TM, thylakoid membrane.

The RubisCO enzyme of cyanobacteria, the only prokaryotes capable of performing oxygenic photosynthesis, exhibits a higher maximal reaction velocity than its plant homologs, but retains a low specificity for CO_2_ rendering it inefficient under present‐day atmospheric CO_2_ concentrations (Price et al., 2008). To overcome this limitation, a sophisticated CCM has evolved in cyanobacteria that improves conditions for the carboxylation reaction (Badger & Price, 2003; Price et al., 2008). This mechanism comprises several Ci transport systems that actively elevate intracellular Ci concentration relative to extracellular levels. Ci uptake is complemented by carboxysomes, proteinaceous microcompartments analogous to algal pyrenoids, that encapsulate RubisCO together with carbonic anhydrase (CA) (Figure 1). Because the cytoplasm of most cyanobacterial species lacks CA, accumulated HCO_3_^−^ is not converted to CO_2_ and remains trapped in the cell as its negative charge reduces passive diffusion across the plasma membrane (Rae et al., 2013). Cytoplasmic HCO_3_^−^ then diffuses into the carboxysome, where CA catalyzes the reversible interconversion of HCO_3_^−^ and CO_2_ (Rowlett, 2010). The generated CO_2_ is retained in the carboxysome, where it is rapidly fixed by RubisCO, thereby driving the CA reaction towards CO_2_ production. The resulting carbon assimilates subsequently diffuse out of the carboxysome and enter the cytoplasmic CBB cycle (Tsai et al., 2007).

### Two types of carboxysomes are found in cyanobacteria

Carboxysomes are well‐investigated members of the broader family of bacterial microcompartments (BMCs), protein‐based organelles that compartmentalize specific metabolic pathways in bacteria. BMCs contribute to environmental adaptation by segregating enzymes from potentially toxic intermediates while permitting the selective exchange of required metabolites (Faulkner et al., 2017; Mahinthichaichan et al., 2018). Despite their functional diversity, all BMCs share a conserved architectural principle: they are entirely proteinaceous, lack a lipid bilayer, and form polyhedral shells that act as diffusion barriers (Axen et al., 2014; Kerfeld et al., 2018).

Carboxysomes, the first BMCs to be discovered (Niklowitz & Drews, 1956), are present in all cyanobacteria as well as in several chemoautotrophic proteobacteria (Correa et al., 2025; Erwin et al., 2012; Kerfeld & Melnicki, 2016; Liu, 2022; Rae et al., 2013). These polyhedral compartments, typically 100–400 nm in diameter (Kerfeld et al., 2018; Rae et al., 2012, 2013), encapsulate RubisCO together with a CA inside a structurally virus‐like shell (Kerfeld & Melnicki, 2016; Tsai et al., 2007). The shell contains highly conserved charged pores that are likely crucial for metabolite exchange, allowing for selective import of HCO_3_^−^, while restricting the passage of nonpolar CO_2_ and O_2_ (Faulkner et al., 2017; Kinney et al., 2011; Mahinthichaichan et al., 2018). However, recent computational simulations suggested that the shell is also permeable to small nonpolar molecules such as CO_2_ and especially O_2_, at the interface of shell proteins; nevertheless, the net effect of CO_2_ elevation can competitively inhibit the oxygenase reaction of RubisCO (Nguyen et al., 2026; Sarkar et al., 2024). In the lumen, CA catalyzes the interconversion of HCO_3_^−^ to CO_2_, thereby elevating the CO_2_ concentration around RubisCO and enhancing carboxylation efficiency despite the high O_2_ permeability of the carboxysome membrane (Rowlett, 2010). With the meanwhile huge number of fully sequenced cyanobacterial genomes, two distinct carboxysomes, α and β, can be defined, which correlate with the RubisCO isoform present and are encoded primarily by the *cso* and *ccm* operons, respectively (Badger et al., 2002; Badger & Bek, 2008; Rae et al., 2013; Roberts et al., 2012). Although α‐ and β‐carboxysomes share similar morphology and functional principles, they differ markedly in shell protein composition, assembly pathways, and internal organization (Rae et al., 2013; Whitehead et al., 2014).

#### Structure and assembly of α‐carboxysomes

**Figure 2:**
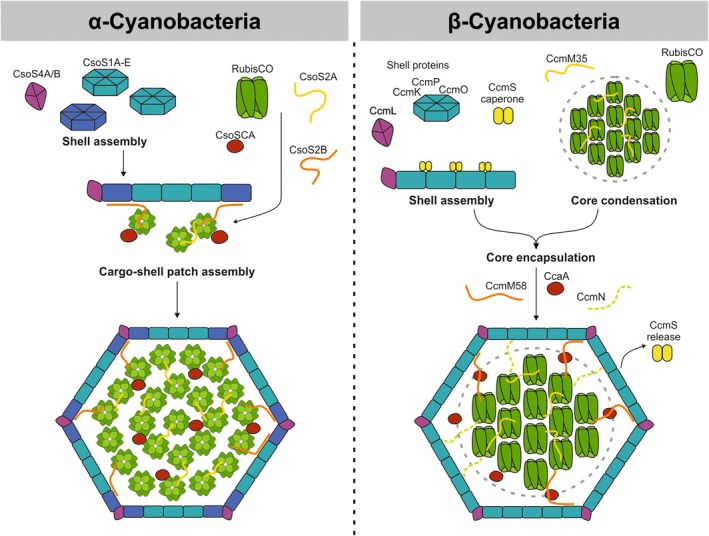
Comparison of α‐ and β‐carboxysome structure and assembly. Although α‐ and β‐carboxysomes perform an equivalent role in carbon fixation by compartmentalizing ribulose‐1,5‐bisphosphate carboxylase/oxygenase (RubisCO) and carbonic anhydrase, they exhibit marked differences in shell protein composition, internal RubisCO packing, and assembly pathways. Assembly of α‐carboxysomes is proposed to follow a pathway, in which initially formed shell patches are subsequently filled with enzymatic cargo. In contrast, for β‐carboxysomes, the core is condensed prior to encapsulation by shell patches. Specific to the assembly of β‐cyanobacteria that encode both CcmS and CcmK1 (such as *Synechocystis* sp. PCC 6803 or *Nostoc* sp. PCC 7120), CcmK possesses a C‐terminal extension that interacts with CcmS (Cheng et al., 2024). Conversely, a large group of β‐cyanobacterial species possess alternative CcmK2 proteins that lack this C‐terminal extension. Lacking one or both of these components, these species are likely employing alternative carboxysome assembly mechanisms.

The phylogenetic group of primarily marine α‐cyanobacteria, including *Prochlorococcus* and marine *Synechococcus* strains, possess α‐carboxysomes. They encapsulate Form 1A RubisCO, composed of large (CbbL) and small (CbbS) subunits, together with β‐class CA (CsoSCA) (Blikstad et al., 2023; Evans et al., 2023; Ni et al., 2022; Pulsford et al., 2024; Rae et al., 2013). The genes encoding core α‐carboxysome components are organized in the *cso* operon, which can also include other CCM genes (Rae et al., 2011; Roberts et al., 2012). The carboxysome shell is constructed from multiple polypeptides containing BMC (Pfam03319) domains (Kerfeld et al., 2010; Kinney et al., 2011). Shell facets are formed by CsoS1A‐E proteins, which assemble into hexamer‐like building blocks, which pack into flat protein sheets and together enclose the microcompartment (Figure 2). Most CsoS1 proteins adopt the so‐called BMC‐H fold and assemble as homohexamers, meaning six identical protein subunits come together to form a hexagonal disk. Other shell proteins contain a BMC‐T domain and assemble as trimers that are structurally arranged to resemble hexamers (‘pseudo‐hexamers’) (Liu, 2022; Ochoa & Yeates, 2021; Tsai et al., 2007, 2009). These hexamers feature centrally located charged pores that mediate import and export of polar or charged metabolites (such as HCO_3_^−^, Mg^2+^, RuBP, and 3‐PGA); however, they also exhibit distinctly different permeabilities to nonpolar molecules such as O_2_ and CO_2_ (Evans et al., 2023; Klein et al., 2009; Mahinthichaichan et al., 2018; Nguyen et al., 2026; Sarkar et al., 2024). For example, CsoS1D forms pseudo‐hexamers that can occupy distinct open and closed states, potentially allowing regulated passage of metabolites, although direct evidence for RuBP transport is lacking (Evans et al., 2023; Klein et al., 2009). In a recent computational simulation of synthetic carboxysomes, Sarkar et al. (2024) further identified the potential for CO_2_ to enter and leak through the carboxysome shell (Sarkar et al., 2024). Notably, their simulations additionally detected an even higher number of O_2_ entry events, primarily occurring at the interfaces between shell proteins. In *Cyanobium* sp. PCC 7001, the shell consists primarily of CsoS1A and CsoS1E hexamers, with CsoS1E surrounding CsoS4A/B pentamers at the vertices (Evans et al., 2023). The 12 vertices of the icosahedral shaped carboxysomes are sealed by pyramidal CsoS4A and CsoS4B pentamers, which are present in all α‐carboxysomes (Evans et al., 2023; Kerfeld et al., 2010; Kinney et al., 2011). Synthetic shells formed *in vitro* demonstrate that CsoS4 pentamers and CsoS1 hexamers can also assemble into hetero‐oligomers, which may modulate shell permeability (Evans et al., 2023; Wang et al., 2024).

The highly conserved and disordered scaffold protein CsoS2 serves as a central hub linking RubisCO and the shell (Cai et al., 2015; Liu et al., 2018; Ni et al., 2022) (Figure 2). While in the γ‐proteobacterium *Halothiobacillus neapolitanus* two CsoS2 isoforms (CsoS2B and the shorter CsoS2A), generated via a programmed ribosomal frameshift, exist (Cai et al., 2015; Chaijarasphong et al., 2016; Oltrogge et al., 2020), many other bacteria including cyanobacteria, such as *Cyanobium* sp. PCC 7001, have only a single CsoS2 isoform, which allowed engineering carboxysome into plant chloroplast (Long et al., 2018; Ni et al., 2022). CsoS2 is organized into N‐terminal, middle, and C‐terminal regions, each containing multiple repeats of conserved motifs that mediate interactions with their respective binding partners (Cai et al., 2015; Chaijarasphong et al., 2016; Oltrogge et al., 2020). The central CsoS2 domain interacts with joint of three connected shell hexamers and likely stabilizes their interaction (Oltrogge et al., 2020; Zhou et al., 2024). The number of repeats in this region thereby determine the carboxysome size (Oltrogge et al., 2024; Zhou et al., 2024). The C‐terminal region anchors to the shell vertices and likely assists in full encapsulation of the nascent carboxysome (Cai et al., 2015; Oltrogge et al., 2020). The N‐terminal domain has four repetitive motifs, separated by flexible linkers that engage at both large and small subunits of RubisCO via salt bridges and cation‐π interactions (Chaijarasphong et al., 2016; Oltrogge et al., 2020). Ni et al. (2022) propose two modes of CsoS2‐RubisCO interaction: multiple CsoS2 molecules binding multiple RubisCO units, forming a network, or one‐to‐one binding. The network model is considered more likely as it is supported by *in vitro* observations (Ni et al., 2022). Evans et al. additionally showed that not only CsoS2 connects RubisCO but RubisCO‐RubisCO interactions also occur both within and between layers via CbbL loop regions and CbbS helices respectively (Evans et al., 2023).

RubisCO packing within α‐carboxysomes is heterogeneous and species‐specific. In *Prochlorococcus*, RubisCO forms three concentric layers (Zhou et al., 2024), while in *Cyanobium* sp. PCC 7001 three (Ni et al., 2022) or four layers are observed (Evans et al., 2023). Zhou et al. analyzed the organization of RubisCO in *Prochlorococcus* α‐carboxysomes in more detail, finding 72 enzymes in the outer layer (1 at each of the 12 vertices and 3 at each of the 20 facets) and 32 in the middle layer (1 at each vertex and 1 at each facet). They subsequently proposed a general formula to calculate the number of RubisCO per layer (*N*): *N* = 12 + 20 × 3^*n*−2^ (*n* ≥2), where n denotes the layer number from the center outward (Zhou et al., 2024). To supply RubisCO with substrate, the carboxysomal lumen contains a second enzyme, CsoSCA (formerly known as CsoS3), a β‐class CA that facilitates interconversion of accumulated bicarbonate and CO_2_ (Heinhorst et al., 2006). Recent structural and functional investigations revealed that CsoSCA assembles into a hexameric trimer of dimers; furthermore, it is allosterically activated by the RubisCO substrate RuBP and is subject to redox regulation (Pulsford et al., 2024; Vogiatzi et al., 2026).

Assembly of α‐carboxysomes is proposed to follow an ‘outside‐in’ pathway (Blikstad et al., 2023; Evans et al., 2023; Metskas et al., 2022; Ni et al., 2022; Oltrogge et al., 2020). In this model, hexameric shell proteins first assemble into patches that are stabilized by CsoS2 via its central and C‐terminal regions, while the N‐terminus recruits RubisCO. Multiple shell‐cargo patches then merge, forming a complete icosahedral shell with vertices closed by CsoS4A/B pentamers (Figure 2). Although most assembly studies rely on recombinant shell expression in *Escherichia coli*, observations in native systems such as *H*. *neapolitanus*, *Prochlorococcus*, and *Cyanobium* provide important insights into the interplay between shell proteins, scaffolds, and enzymatic cargo. Despite these advances, the precise molecular details of α‐carboxysome biogenesis remain to be fully elucidated (Oltrogge et al., 2024; Rae et al., 2011). Also, it is worthwhile to mention that the structural studies on *H*. *neapolitanus* CA, CsoSCA, revealed that CsoSCA interacts directly with the CsoS1A shell hexamer and with Rubisco via an intrinsically disordered N‐terminal domain (Blikstad et al., 2023; Ng et al., 2025).

#### Structure and assembly of β‐carboxysomes

β‐Carboxysomes are found exclusively in β‐cyanobacteria, including *Synechocystis* sp. PCC 6803, *Synechococcus elongatus*, and *Lyngbya* species, and are seemingly absent in all other bacteria (Stanier et al., 1979). They encapsulate Form 1B RubisCO, composed of large (RbcL) and small (RbcS) subunits as well as one or two types of carbolic anhydrases (Price et al., 1998). Beta‐carboxysomes are localized throughout the cell and can potentially interact with the cytoskeleton, as observed in *Anabaena* (Savage et al., 2010). The core β‐carboxysome genes are encoded within the *ccm* operon, whereas other associated genes are distributed throughout the cyanobacterial genome. Notably, *ccaA*, which encodes a β‐class CA, is never part of the original operon, and *rbcLXS* genes are rarely found in the core operon (Alm et al., 2005). The β‐carboxysome is a multilayered protein structure with selective diffusion pores that allow import and export of specific charged or polar metabolites (Faulkner et al., 2020; Kinney et al., 2011; Sommer et al., 2019), while small, uncharged CO_2_ and O_2_ diffuse though the shell at the protein–protein interfaces (Sarkar et al., 2024). The outer shell consists primarily of proteins containing the BMC (Pfam03319) domain, including multiple paralogs of CcmK (1–4, not all paralogs present in all β‐cyanobacteria) (Kinney et al., 2011; Sommer et al., 2019), CcmL (Tanaka et al., 2008, 2009), and CcmO (Kerfeld et al., 2010) (Figure 2).

CcmK proteins assemble into flat hexameric tiles that form the facets of the icosahedral shell (Cannon et al., 2010; Tsai et al., 2009). The CcmK hexamer features a central, charged pore formed by highly conserved residues (e.g., KIGS in CcmK2 and RAGS in CcmK4) from each monomer, that likely mediate the passage of negatively charged or polar metabolites (Faulkner et al., 2020; Kinney et al., 2011). The isoform CcmK2 is essential for shell formation, while CcmK3 and CcmK4 are likely not essential for carboxysome biogenesis but could contribute to subcellular localization and full shell function (Sommer et al., 2019). Sommer et al. further observed hetero‐hexamer formation between CcmK1/K2 and CcmK3/K4 paralogs, which likely modulates shell permeability (Sommer et al., 2019). Additionally, structural studies resolved stacked dodecameric CcmK2/K3/K4 assemblies, suggesting that the shell facets could form a double layer, however such structures have not yet been found *in vivo* (Samborska & Kimber, 2012; Sommer et al., 2019). Similar to CsoS4A/B proteins in α‐carboxysomes, CcmL forms pentagonal pyramidal pentamers that occupy the vertices of the icosahedral shell (Tanaka et al., 2008, 2009). CcmO, a protein that is also essential for carboxysome formation, potentially surrounds CcmL at vertices and connects the shell facets (Rae et al., 2012). No structure for the protein has yet been published, as crystallization has proven to be difficult, but it is predicted to form trimers (Sommer et al., 2017). CcmP, a homolog of the α‐carboxysomal CsoS1D, forms dimers of trimers and contains a central pore with a binding pocket for 3‐PGA, facilitating metabolite transport (Cai et al., 2013).

A subsequent inner layer, containing scaffold proteins CcmM and CcmN connects the shell to the carboxysomal lumen (Faulkner et al., 2017; Kinney et al., 2012; Long et al., 2011). CcmM acts as a central scaffold, analogous to CsoS2 in α‐carboxysomes, and is crucial for carboxysome assembly and function (Faulkner et al., 2017; Long et al., 2010; Zang et al., 2021). The gene *ccmM* produces two isoforms from a single transcript with an internal ribosomal binding site: CcmM‐58 (full‐length) and CcmM‐35 (short), with the short form being more abundant (Long et al., 2007, 2010). Both isoforms are essential for carboxysome formation, as deletion of *ccmM* prevents carboxysome biogenesis entirely (Ludwig et al., 2000). CcmM‐58 localizes beneath the outer shell, bridging the shell to lumen proteins (Kinney et al., 2012; Long et al., 2010). Its N‐terminal γ‐class CA‐like domain mediates trimerization and additional interactions with CcaA, CcmN, and potentially CcmK1, K2, K4, and CcmL (Long et al., 2007, 2010; Zang et al., 2021). The C‐terminal domain contains 3–5 RubisCO small subunit‐like (SSU‐like) domains (~85 aa each, separated by ~40 aa hydrophilic linkers) with ~30% sequence identity to RbcS, which bind fully assembled RubisCO holoenzymes via the cleft between RbcL dimers, forming higher‐order condensates (Blikstad et al., 2023). CcmM‐35 contains only SSU‐like domains and binds only fully assembled RubisCO in the lumen, thereby preventing premature encapsulation of chaperone‐bound or octameric RbcL (L_8_) intermediate complexes (Long et al., 2007, 2010, 2011; Wang, Sun, et al., 2019). CcmN additionally acts as an adaptor connecting outer shell layers with the RubisCO core, with its N‐terminal domain interacting with CcmM and the C‐terminal domain binding CcmK2 (Kinney et al., 2012). Recent work shows CcmM and CcmN can form heterotrimers (1 CcmM + 2 CcmN) that potentially recruit the shell to the RubisCO core (Sun et al., 2021). In β‐carboxysome assembly, the scaffolding protein CcmM mediates the formation of a pro‐carboxysome biomolecular condensate containing RubisCO, CA, and the shell adaptor CcmN. Recent work reveals that CcmN is recruited to the condensate's periphery as a hetero‐complex (comprised of three CcmN protomers and one CcmM protomer) to ensure that shell formation and maturation proceed only after the internal RubisCO/CA enzymes have been assembled (Zang et al., 2026). Interestingly, CO_2_ concentration was found to alter the redox state and encapsulation of cyanobacterial carboxysomes. Under high CO_2_ (3%), pro‐carboxysome‐like structures formed with partially assembled shells that failed to fully encapsulate RubisCO and form closed microcompartments, exposing their contents to the cytosol (Huffine et al., 2026).

β‐Cyanobacteria harbor two types of carboxysomal CAs: the β‐class CcaA and the γ‐CA‐like domain of CcmM‐58 (Klanchui et al., 2017; Peña et al., 2010). Some species possess only one active CA, whereas others retain both (Kimber, 2014; Klanchui et al., 2017; Peña et al., 2010). CcaA, which is essential in *Synechocystis* PCC 6803 and *S. elongatus*, where CcmM‐58 lacks CA activity (So et al., 2002), is hypothesized to have originated in *Cyanothece* and spread via horizontal gene transfer (Peña et al., 2010). Functional CcaA, that assembles into trimers of dimers via its extended C‐terminus (So et al., 2002), shows relatively low activity which is still sufficient to saturate RubisCO with CO_2_ (McGurn et al., 2016). It localizes at the inner side of the carboxysome shell (Faulkner et al., 2017) where it has been found to form complexes with RubisCO (Long et al., 2007). CcaA is redox‐regulated with bicarbonate dehydration activity restricted to the carboxysome (Peña et al., 2010). In the cytoplasm, CcaA is inactivated and degraded to prevent interference with the CCM (Rae et al., 2013).

The N‐terminal γ‐CA‐like domain of CcmM‐58 is likely the ancestral CA in β‐carboxysomes, as it is present in all species regardless of CcaA (Peña et al., 2010). This hypothesis is further supported by the fact that *ccaA* is not part of *ccm*, the core operon encoding for β‐carboxysomes, whereas *ccmM* is (Alm et al., 2005). In organisms that also encode CcaA, this domain is mostly inactive and is only responsible for shell‐lumen crosslinking (Cot et al., 2008; Kimber, 2014). The N‐terminal CA activity was first demonstrated in *Thermosynechococcus elongatus* BP‐1, which lacks CcaA (Peña et al., 2010). Their CA‐like domain shares 35% sequence identity with the γ‐class CA of *Methanosarcina thermophilus* and contains a redox‐sensitive disulfide bond (Cys194–Cys200), inactive in the reducing cytoplasm but functional in the oxidizing environment of the carboxysome (Peña et al., 2010).

The lumen of β‐carboxysomes contains L_8_S_8_ RubisCO holoenzymes (a hexadecamer consisting of 8 RbcL and 8 RbcS of ~530 kDa), scaffolded by CcmM‐35 into three‐dimensional paracrystalline arrays (Long et al., 2007, 2010). A recent study suggested that RubisCO and CcmM might interact dynamically to form a liquid‐like matrix via liquid–liquid phase separation, rather than the rigid arrays previously proposed (Wang, Yan, et al., 2019). However, the cryo‐electron tomography has revealed a unique packaging pattern within the β‐carboxysome, featuring three distinct arrangements between adjacent RubisCO molecules: head‐to‐head, head‐to‐side, and side‐by‐side. The RuBisCOs in the outer layer are regularly aligned along the shell, with the majority interacting directly with the shell (Kong et al., 2024). Ultimately, RubisCO and subsequent carboxysome biogenesis is a complex process that relies on the assistance of multiple chaperones (Li et al., 2022). Folding of individual RbcL monomers is mediated by the chaperone complex GroEL‐GroES, which are subsequently replaced by the chaperone RbcX and RubisCO assembly factor Raf1 (Liu et al., 2010; Saschenbrecker et al., 2007; Xia et al., 2020). Together, they facilitating the stepwise formation of L_8_ cores (Hauser et al., 2015; Liu et al., 2010; Saschenbrecker et al., 2007; Xia et al., 2020). Homodimeric Raf1 predominantly acts at early stages, interacting with antiparallel RbcL dimers (Xia et al., 2020), while RbcX assists in later assembly by binding the C‐terminal motif of RbcL (Bracher et al., 2011). Cryo‐EM structures revealed that Raf1 facilitates RubisCO assembly by mediating RbcL dimer formation and dimer–dimer interactions. Consequently, the Δ*raf1* mutant is unable to form intact carboxysomes; instead, it generates numerous intermediate assemblies—comprising RubisCO, CcaA, CcmM, and CcmN—without shell encapsulation. Additionally, it produces a low abundance of carboxysome‐like structures characterized by reduced dimensions and irregular shell shapes (Huang et al., 2020). RbcX has further been observed to catalyze RubsiCO condensate dissolution, potentially a mechanism to regulate RubisCO and carboxysome biogenesis (Li et al., 2022). Capping of the RbcL core by RbcS then displaces Raf1 and RbcX to complete holoenzyme formation (Hauser et al., 2015; Liu et al., 2010).

Subsequent β‐carboxysome assembly is suggested to follow an ‘inside‐out’ pathway, with the RubisCO matrix forming first, followed by its encapsulated with the carboxysome shell. Initially, CcmM‐35 binds assembled RubisCO and establishes the condensate that functions as the carboxysome nucleus (Long et al., 2010; Wang, Sun, et al., 2019; Zang et al., 2021). Cheng et al. (2024) suggested that simultaneously, patches of carboxysome shell building blocks consisting of CcmK1 hexamers and additional shell proteins (e.g., CcmL, CcmO, and CcmP). For some, this patch formation is assisted by the dimeric chaperone CcmS, which interacts specifically with the C‐terminus of CcmK1 (Figure 2) (Chen, Zheng, et al., 2023). However, as not all cyanobacteria contain CcmS and/or CcmK1, alternative mechanisms for carboxysome assembly must exist. CcmN and CcmM‐58 are then recruited to the completed RubisCO–CcmM‐35 core structure where they initiate its encapsulation with the shell patches (Figure 2). Following completion of the shell, CcmS dissociates into the cytoplasm, although the precise mechanistic details of this process remain unresolved (Cheng et al., 2024).

### Five Ci uptake systems have been identified in cyanobacteria

Together with carboxysomes, Ci transporters form the highly effective cyanobacterial CCM. In model β‐cyanobacteria, such as *Synechocystis* sp. PCC 6803, five protein complexes are currently known to be involved in the enrichment of HCO_3_^−^ in the cytoplasm (Price, 2011) (Figure 2). NDH‐1_3_ and NDH‐1_4_ also referred to as CO_2_‐hydrating photosynthetic complexes PC1 and PC2 (Hagemann & Kaplan, 2020), are localized in the thylakoid membrane, where they catalyze the conversion and hydration of CO_2_ to HCO_3_^−^ (Ohkawa et al., 2001; Sun et al., 2019; Zhang et al., 2004). In contrast, the HCO_3_^−^‐transporters BCT1 (Omata et al., 1999), SbtA (Shibata et al., 2002), and BicA (Price et al., 2004) are embedded in the cytoplasmic membrane and actively import HCO_3_^−^ into the cell. Notably, α‐cyanobacteria encode a more limited set of Ci transporters, typically lacking the high‐affinity BCT1 and NDH‐1_3_ systems (Cabello‐Yeves et al., 2022; Rae et al., 2013). Ci transporters can further be classified based on substrate affinity and transcriptional regulation: the genes of low‐affinity, high‐flux systems such as BicA and NDH‐1_4_ are constitutively expressed (Price et al., 2004; Shibata et al., 2001; Wang et al., 2004), whereas expression of genes corresponding to high‐affinity, low‐flux transporters including SbtA, BCT1, NDH‐1_3_ is induced only under Ci‐limiting (LC) conditions (Wang et al., 2004). Several comprehensive reviews addressing different aspects of cyanobacterial Ci uptake systems and regulation have been published in recent years (for additional insights see, Kupriyanova et al., 2023; Rottet et al., 2021; Kurkela & Tyystjärvi, 2024).

#### Components and mechanisms of bicarbonate uptake in cyanobacteria

##### BCT1 complex

The BCT1 transporter, initially identified through its 42 kDa periplasmic substrate‐binding protein CmpA, was the first Ci transporter discovered and characterized in cyanobacteria (Omata & Ogawa, 1986). It is a primary active, high‐affinity HCO_3_^−^ uniporter that belongs to the ATP‐binding cassette (ABC) transporter family, also known as traffic ATPases (Omata et al., 1999). BCT1 is widespread among cyanobacteria, occurring in both α‐ and β‐cyanobacteria (Cabello‐Yeves et al., 2022; Rae et al., 2011; Sandrini et al., 2014). Rae et al. showed that *Synechococcus* WH5701 acquired the genes encoding for the transporter from β‐cyanobacteria through horizontal gene transfer (Rae et al., 2011), the same might also apply to additional α‐cyanobacterial lineages (Cabello‐Yeves et al., 2022). BCT1 supports a medium bicarbonate flux and exhibits high affinity toward its substrate, with reported *K*_D_ values of ~15 μM when overexpressed in cyanobacteria (Omata et al., 2002) and as low as ~5 μM when recombinantly expressed in *E. coli* (Maeda et al., 2000). The heteromeric, multi‐subunit transporter is encoded by the *cmpABCD* operon, that shares high homology with the *nrtABCD* operon encoding the nitrate/nitrite ABC transporter, where NrtA acts as the substrate‐binding protein. Expression of *cmpA* and the entire operon is induced under Ci‐limited conditions (Omata et al., 1999), but only in light (McGinn et al., 2003).

**Figure 3:**
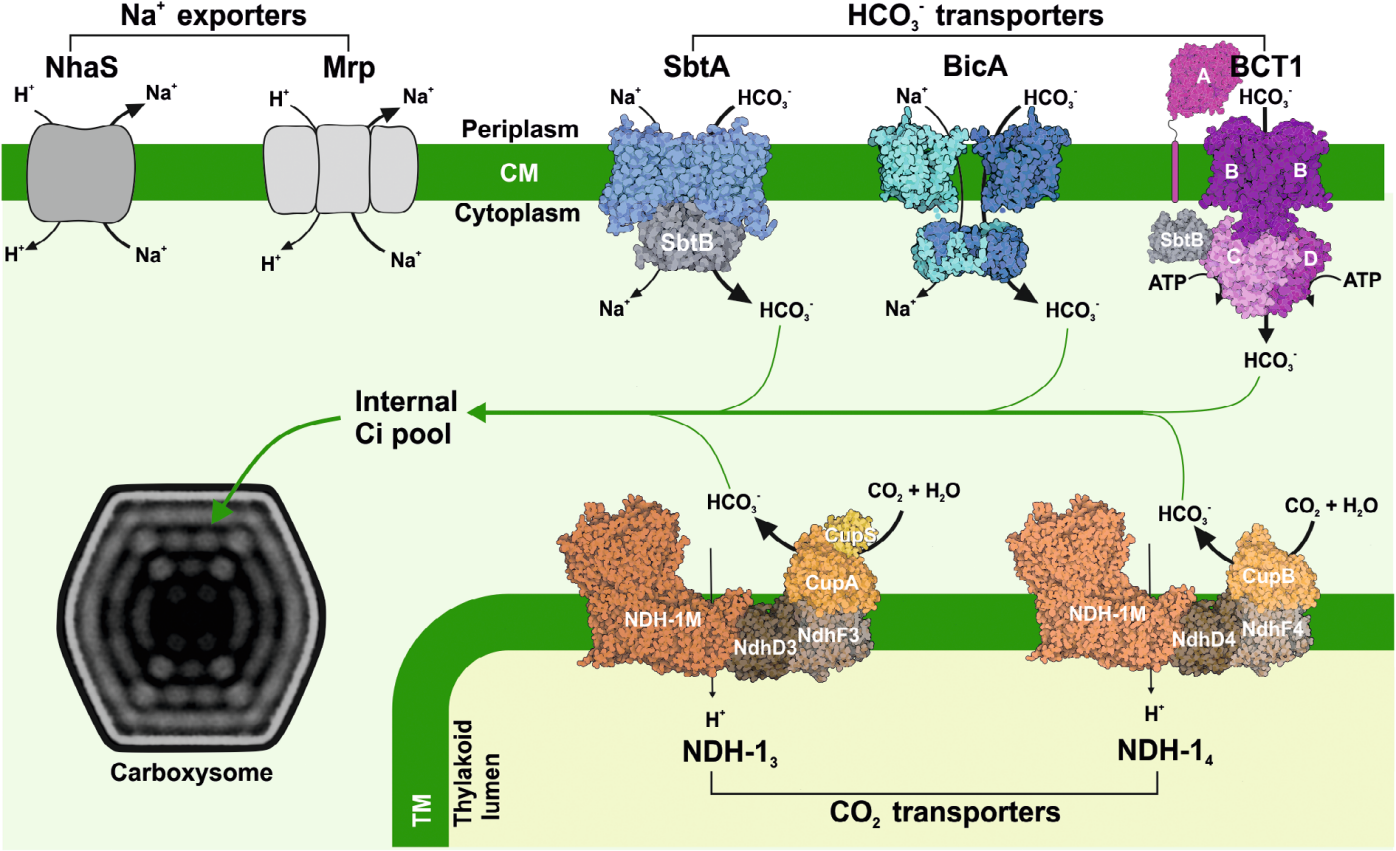
Mechanisms of inorganic carbon (Ci) uptake in cyanobacteria. Five distinct Ci transport systems mediate the import of bicarbonate (HCO_3_^−^) and CO_2_ into the cell, collectively contributing to increasing the intracellular Ci pool that supplies the carboxysome. Sodium‐dependent bicarbonate transporters SbtA and BicA facilitate the symport of HCO_3_^−^ and Na^+^ across the cytoplasmic membrane, with dedicated Na^+^‐exporters (NhaS1‐6 and Mrp Systems) maintaining Na^+^ and ionic homeostasis. The multimeric ABC transporter BCT1 (CmpABCD complex) enables ATP‐dependent uptake of HCO_3_^−^ (Li et al., 2026). SbtB, the master cyanobacterial carbon control protein, interacts with all HCO_3_^−^ uptake systems (SbtA, BicA, and CmpC of the BCT1 complex) to regulate the HCO_3_^−^ flux into the cell (Haffner et al., 2025). SbtB acts as a valve plug on SbtA to prevent the backward flux of HCO_3_^−^ through the SbtA tunnel, while its exact regulatory modes on BicA and CmpABCD complex remain unclear. SbtB may function as an allosteric inhibitor of CmpBCD, similar to how PII regulates nitrate transporter by forming a PII‐NtrC inhibitory complex. In support of this, SbtB interacts with CmpC subunit (Haffner et al., 2025); this interaction was modeled based on the closely related NrtABCD‐PII complex (Li et al., 2024). In addition, the thylakoid membrane‐associated multi‐subunit NDH‐1_3_ and NDH‐1_4_ complexes promote the hydration of cytosolic CO_2_ to HCO_3_^−^ and thereby further contribute to the cytosolic Ci pool, which becomes available for carbon fixation after carboxysomal conversion to CO_2_ by carbonic anhydrase as shown in (Figure 1).

The functional transporter consists of four different subunits: CmpA, CmpB, CmpC, and CmpD (Koropatkin et al., 2007) (Figure 3). CmpA serves as the substrate‐binding lipoprotein; it is attached to the periplasmic side of the membrane via a lipid anchor. Additional structural work by Koropatkin et al. showed that CmpA binds carbonic acid, the fully protonated form of HCO_3_^−^ that is present at pH 5, near the entrance of its C‐clamp‐shaped binding cleft, while HCO_3_^−^ binds deeper in the pocket, where Glu271 stabilizes the negatively charged oxygen and also acts as an acceptor for the hydrogen bond of the protonated oxygen. Surprisingly, the crystal structure of the transporter also revealed a Ca^2+^ ion coordinated by the substrate‐binding site residues Glu271, Gln155, and Gln198. Subsequent experiments showed that HCO_3_^−^ and Ca^2+^ bind cooperatively with no HCO_3_^−^ binding occurring in the absence of Ca^2+^. The physiological relevance of Ca^2+^ is not fully understood yet, as of now there is no evidence for Ca^2+^ co‐transport. It may instead enhance substrate selectivity/affinity or it simply is an artifact of recombinant expression or protein purification (Koropatkin et al., 2007). The functional HCO_3_^−^ uniporter further consists of the likely homodimeric membrane integral protein CmpB that forms the substrate transport channel, and the cytoplasmic ATPases CmpC and CmpD that catalyze ATP hydrolysis. Analogous to NrtC, CmpC contains an additional C‐terminal region homologous to CmpA, which may function as a regulatory solute‐binding domain (Koropatkin et al., 2007).

As the native BCT1 shows no activity when expressed in *E. coli*, it was hypothesized that additional post‐translational modifications or modifications in the regulatory C‐terminal domain of the CmpC subunit may be required for transporter function (Rottet et al., 2024). Since *E. coli* lacks a cyanobacterial CCM and the associated LC‐dependent regulatory network, these modifications may not occur in this heterologous system. Consistent with this idea, several LC‐induced phosphorylation sites were identified in CmpA (Ser110, Thr129) and CmpB (Thr3), pointing to a possible mechanism for rapid activation during Ci limitation (Spät et al., 2021). Nevertheless, engineering efforts successfully evolved new, constitutively active BCT1 forms that were used to target plant chloroplasts (Rottet et al., 2024). The precise transport mechanism of BCT1 specifically has not been fully understood yet. However, it is expected to follow the general alternating‐access cycle typical of ABC importers (described e.g. by Lewinson and Livnat‐Levanon (2017) and Wilkens (2015)). In this model, the substrate‐binding domain (CmpA) first captures the substrate (HCO_3_^−^) in the periplasm and transfers it to the outward‐facing transmembrane (TM) domains (CmpB). ATP binding to the nucleotide binding domains (NBDs of CmpC and CmpD) induces NBD dimerization and subsequent ATP hydrolysis, which switches the transporter into an inward‐facing state and releases bicarbonate into the cytoplasm. The substrate, ADP and inorganic phosphate are then released into the cytoplasm and the is reset to its initial conformation. However, as ABC transporters likely do not all follow an identical mechanism, the specific mechanistic details and the precise order in which the steps of their substrate transport cycle occur still need to be determined (Wilkens, 2015). The first structural insights into the transport activity of the BCT1 complex revealed a surprising mechanism (Li et al., 2026). The ATPase subunit CmpC, which possesses a C‐terminal regulatory domain (CRD), was found to sense nitrate levels, thereby coupling HCO_3_^−^ transport to nitrogen availability. At low nitrate levels, CmpBCD complex adopts an inhibited conformation in which the CRD blocks the NBDs of both CmpC and CmpD subunits. Upon binding nitrate to CRD, it becomes highly flexible and is released from the NBDs, thus restoring the HCO_3_^−^ transport of BCT1 complex (Li et al., 2026). Hence, further investigations are needed to fully understand the transport mechanism of the BCT1 complex for engineering purposes.

##### SbtA

The sodium‐dependent bicarbonate transporter SbtA acts as symporter, coupling Na^+^ translocation to HCO_3_^−^ uptake against its concentration gradient (Figure 3). It was first characterized in *Synechocystis* sp. PCC 6803, now known as SbtA1 (Shibata et al., 2002). It belongs to the wider sodium solute symporter (SSS) family, classified as TC.2.A.83 in the Transporter Classification Database (Price, 2011). Functionally, SbtA is a high‐affinity, low‐flux bicarbonate transporter (Price et al., 2004), exhibiting *K*_m_ values of 2–5 μM in *Synechococcus* sp. PCC 7002 (Price et al., 2004). Transport is Na^+^‐dependent (Price et al., 2002; Shibata et al., 2002), requiring approximately 1–15 mM Na^+^ for half‐maximal activity (Du et al., 2014). The degree of Na^+^ sensitivity varies among species and is partly shaped by their habitat, with euryhaline or marine cyanobacteria such as *Synechococcus* sp. PCC 7002 showing higher Na^+^ requirements (Du et al., 2014). In *Synechocystis* sp. PCC 6803, SbtA1 is a 40 kDa protein localized to the cytoplasmic membrane (Zhang et al., 2004). Early SDS‐PAGES suggested a tetrameric complex based on an apparent mass of ~160 kDa; however, this was later attributed either to a heterohexameric assembly of three SbtA and three SbtB subunits. Recent cryo‐EM structures unambiguously demonstrate that the functional transporter forms a homotrimer (Fang et al., 2021; Liu et al., 2021). SbtA is widely distributed across both α‐ (Du et al., 2014; Price et al., 2008) and β‐cyanobacteria (Price et al., 2002; Shibata et al., 2002), though its presence is not universal. SbtA also occurs in a limited number of non‐cyanobacterial taxa, for example, it shows low‐level sequence similarity to the arabinose transporter AraH of the ABC superfamily (Von Rozycki et al., 2004). In *Synechocystis* sp. PCC 6803, SbtA1 is encoded by *slr1512*, and its transcription is strongly induced under LC conditions (Shibata et al., 2002; Zhang et al., 2004). Interestingly, a new family of SbtA proteins, now known as SbtA2 family, was recently been identified from marine *Synechococcus*. When assessed in *E. coli*, these SbtA2 transporters exhibited high Ci uptake flux and intermediate HCO_3_^−^ affinity (*K*_m_ ≈150 μM), but displayed chloride dependency (Cabello‐Yeves et al., 2022; Rae et al., 2011; Rourke et al., 2026a). Structurally, SbtA2 transporters likely share a similar fold to SbtA1.

The canonical SbtA1 membrane topology that was initially predicted (Von Rozycki et al., 2004), has shown to be consistent with later determined experimental structures (Price, 2011). Price et al. used topology mapping to reveal 10 TM helices arranged as two inverted repeats of five helices (5 + 5) connected by a large intracellular loop (Price, 2011; Price & Howitt, 2014). This arrangement is characteristic of Na^+^‐coupled transporters (Krishnamurthy et al., 2009), surprisingly however, in SbtA both the N‐ and C‐termini are oriented toward the extracellular space (Price, 2011). Detailed cryo‐EM structures further revealed two distinct functional domains: a core (transport) domain composed of TM3‐5 and TM8‐10, and a gate (scaffold) domain formed by TM1‐2 and TM6‐7 (Liu et al., 2021). These domains interact through a buried hydrophobic interface involving TM4‐5 and TM9‐10 of the core domain and all four helices of the gate domain. The trimeric assembly of functional SbtA is stabilized via interactions mediated primarily by the gate domain (Fang et al., 2021; Liu et al., 2021).

Bicarbonate transport by SbtA1 likely follows and elevator‐type alternating‐access mechanism (Fang et al., 2021; Liu et al., 2021). In this model, SbtA1 is initially in an outward‐open conformation, so that both substrates HCO_3_^−^ and Na^+^ can bind to the core domain. Consequently, the core domain becomes occluded and moves towards the cytosolic side, against the rigid gate domain. An inward‐open conformation is achieved, in which both substrates are released into the cytoplasm. Subsequently, the transporter returns to its outward‐open resting state, ready for the next transport cycle. The regulation of SbtA transport activity is mediated through its interaction with the small trimeric PII‐like protein SbtB (Förster et al., 2023; Haffner et al., 2023; Kaczmarski et al., 2019; Selim et al., 2018, 2023), which directly binds SbtA (Fang et al., 2021; Haffner et al., 2023; Liu et al., 2021), analogous to the regulation of the ammonium transporter AmtB by the PII protein GlnK (Conroy et al., 2007; Haffner et al., 2023; Selim & Alva, 2024). Similarly, SbtA2 was found to interact with another PII‐like protein, named as SbtB2 or SbtB‐like (Rourke et al., 2026a; Selim & Alva, 2024).

##### BicA

The second sodium‐dependent bicarbonate transporter, BicA, was first identified in *Synechococcus* sp. PCC 7002 and functions as a Na^+^‐HCO_3_^−^ symporter, with both substrates likely transported in a 1:1 stoichiometric ratio (Price et al., 2004). Additional homologs of BicA (known as BicA2) with relatively low amino acid similarity (25–50%), have also been identified, particularly in α‐cyanobacterial genomes (Price et al., 2008; Rae et al., 2011). The heterologus expression of *Prochlorococcus* BicA2 mediated Na^+^‐dependent HCO_3_^‐^ uptake in *E. coli*, confirming that BicA2 is a low‐affinity, Na^+^‐dependent bicarbonate transporter (Rourke et al., 2026b). The canonical BicA transporters, known as BicA1, are localized in the cytoplasmic membrane and transports the same substrates as SbtA (Figure 3) but shows no sequence similarity to it. BicA operates as a low‐affinity, high‐flux transporter with a *K*_0.5_ of only 38 μM, as determined in *Synechococcus* PCC 7002, with some strains exhibiting even lower affinity (Price et al., 2004). Transport activity is strictly Na^+^‐dependent, with approximately 1.7 mM Na^+^ required for half‐maximal bicarbonate uptake (Price et al., 2004). BicA belongs to the large and functionally diverse SulP/SLC26 family of anion transporters (TC 2.A.53), that display a wide variety of transport function across plants, bacteria, and animals (Alper & Sharma, 2013; Price & Howitt, 2011). BicA is broadly distributed across all major cyanobacterial clades, including marine *Synechococcus*, *Prochlorococcus*, and freshwater strains such as *Synechocystis* sp. PCC 6803 and *Synechococcus* PCC 7002, and also occurs in some proteobacteria (Price et al., 2004; Shelden et al., 2010). In *Synechocystis* PCC 6803, *bicA* is constitutively expressed (Klähn et al., 2015; Wang et al., 2004), whereas in *Synechococcus* PCC 7002 the gene is strongly induced under Ci limitation (Price et al., 2004; Woodger et al., 2007).

Early structural insights into BicA1 accomplished by Shelden et al. came from topology mapping in *Synechococcus* PCC 7002, which suggested an N‐terminal membrane domain containing 12 TM and a cytoplasmic C‐terminal STAS (sulfate transporter and anti‐sigma factor antagonist‐like) domain (Shelden et al., 2010). The name of the STAS domain originates from the determined low sequence similarity to the bacterial anti‐sigma antagonist SpoIIAA (Aravind & Koonin, 2000) and, along with a large cytoplasmic loop between TM8 and TM9, was proposed to play regulatory roles (Shelden et al., 2010). Both N‐ and C‐termini were identified to reside in the cytoplasm. Based on resolved structures of other transporters in the family (e.g., uracil transporter UraA [Lu et al., 2011], rat prestin rPres [Gorbunov et al., 2014]), Price and Howitt speculated, that the BicA1 N‐terminus might instead consist of 14 TM that adopt a 7 + 7 inverted repeat topology (Price & Howitt, 2014). They further proposed that TM 9 and 10 might be missing from the earlier study due to interference of the fusion protein, that is necessary for topology mapping, with native folding and localization of BicA1. This updated 14 TM architecture was confirmed by the crystal structure of *Synechocystis* PCC 6803 BicA1 solved by Wang, Sun, et al. (2019). In this structure, BicA1 adopts the canonical SLC26 fold with 14 TMs organized as a 7 + 7 inverted repeat (TM1‐7 and TM8‐14). TMs 3 and 7 extend only halfway across the membrane, terminating in random coiled coils, that crossover in the middle of the protein. The TM region of BicA is divided into a core/transport domain (TM1‐4 and TM8‐11) and a rigid gate/scaffold domain (TM5‐7 and TM12‐14). The gate domain forms an inverted‐V shape, and a hydrophilic cavity at the core‐gate interface constitutes the conserved substrate‐binding site, formed by TM3, TM8, and TM10. HCO_3_^−^ is coordinated by Thr69 (TM3), Ala301 (TM10), and Na^+^, residues Asp258 and Thr262 (TM8) and Gly300 and Thr302 (TM10) coordinate and Na^+^ (Chan et al., 2025; Wang, Sun, et al., 2019). Wang, Sun, et al. (2019) and Wang, Yan, et al. (2019) further determined that the C‐terminal STAS domain (residues 409–564) is essential for transport function and BicA dimerization (Wang, Sun, et al., 2019).

Similar to the canonical SbtA1, BicA1 operates via an alternating‐access, elevator‐type transport mechanism (Chan et al., 2025; Wang, Sun, et al., 2019), in which the transport domain undergoes both translational and rotational movements relative to the rigid scaffold domain, translocating HCO_3_^−^ across the membrane (Garaeva & Slotboom, 2020). Molecular dynamics simulations have recently been used to model these conformational changes, revealing that both protomers of the homodimeric BicA can undergo the major structural transition between outward‐ and inward‐facing states during transport simultaneously or independently, with the non‐simultaneous transitions being slightly favored (Chan et al., 2025). Closure of the transport site in each conformation state appears to be mediated by hydrophobic residues located at the gate‐core interface, stabilizing the alternating‐access cycle (Chan et al., 2025). These mechanistic insights are solely derived from MD simulations and require further experimental verification.

In order to function efficiently, both Na^+^‐dependent bicarbonate transporters SbtA and BicA require an inward‐directed Na^+^ electrochemical gradient as energy source to drive HCO_3_^−^ uptake into the cell. To maintain this gradient and avoid Na^+^‐oversaturation of the cytoplasm, cyanobacteria must continuously export Na^+^ via Na^+^‐exporters (Figure 3) such as Na^+^/H^+^ antiporters (Tsujii et al., 2024; Wang et al., 2002), multi‐subunit Na^+^ antiporters (Hagemann, 2011), and a proposed Na^+^‐ATPase. In the model cyanobacterium *Synechocystis* sp. PCC 6803, multiple Na^+^/H^+^ antiporters have been described, namely NhaS1‐NhaS6 (Tsujii et al., 2024). These NhaS proteins are secondary active transporters in the cytoplasmic and thylakoid membrane that couple Na^+^ efflux to H^+^ import and thereby maintain the Na^+^ gradient and simultaneously influence intracellular pH. Some cyanobacteria also carry large multi‐subunit Na^+^/H^+^ antiporters analogous to the bacterial Mrp (multiple resistance and pH) family. These consist of six to seven hydrophobic subunits that assemble into a complex exporter, which uses proton motive force to export Na^+^ (Hagemann, 2011).

#### Structural and mechanistical insights into CO_2_ transport via specialized NDH‐1 complexes

Cyanobacteria possess four distinct NDH‐1 complexes: NDH‐1L, NDH‐1L', NDH‐1MS (NDH‐1_3_), and NDH‐1MS' (NDH‐1_4_), some of which share conserved subunits with plants. NDH stands for NAD(P)H dehydrogenase‐like complex, reflecting their structural and evolutionary relationship to respiratory complex I located in the mitochondrial inner membrane. The general function of the NDH‐1 complexes is the cyclic electron transport (CET) around photosystem I, as a mechanism to balance the ATP to NADPH ratio, produced during the photosynthetic light reactions. This avoids the over‐accumulation of reducing equivalents, such as reduced ferredoxin (Fd^red^) and NADPH, which would otherwise lead to the formation of reactive oxygen species at enzymes that use these reducing equivalents as electron donors under high‐light conditions (Laughlin et al., 2019; Zhang et al., 2020). In contrast, the thylakoid membrane NDH‐1MS and NDH‐1MS' complexes catalyze the conversion and hydration of CO_2_ to HCO_3_^−^, exhibiting CA‐like activity. NDH‐1MS' is constitutively expressed and has a low‐affinity CO_2_ (*K*_D_ = 10 μM) for CO_2_. NDH‐1MS, on the other hand, is induced under CO_2_‐limitation and has a higher affinity towards CO_2_ (*K*_D_ = 1–2 μM). The hydration reaction effectively traps nonpolar CO_2_ that passively diffuses over the plasma membrane or escapes the carboxysome by converting it into the charged, membrane‐impermeable HCO_3_^−^, thereby maintaining an inward diffusion gradient for CO_2_. All NDH‐1 complexes share a common core NDH‐1M module, consisting of 11 core subunits (NdhA‐K) homologous to those found in respiratory complex I, and are further associated with a variable module differing for each type (Zhang et al., 2020). Additional oxygenic photosynthesis‐specific (OPS) subunits (NdhL‐Q, NdhS, NdhV) contribute to structure and regulatory control. The complex has an L‐shaped architecture with one arm located within the thylakoid membrane and another peripheral arm extending into the cytoplasm.

Structurally, negatively charged Fd^red^ interacts with the NDH‐1 complex at the apex of its peripheral arm through positively charged surface patches. In *T. elongatus*, the OPS‐subunit NdhO and the core subunit NdhI form a potential Fd‐binding site termed O‐site. This site contains several surface‐exposed lysine residues, two in NdhO and three in an extended β‐hairpin of NdhI, supporting their role in Fd binding (Laughlin et al., 2019). Interestingly, however, deletion of *ndhO* increases CET activity, suggesting that NdhO may act as a negative regulator of NDH‐1‐mediated CET via a yet unknown mechanism (Zhang et al., 2020). A second potential Fd‐binding site formed by NdhS, termed S‐site. Its positively charged arginine residues are surface‐exposed in plant NDH‐1 complexes; however, they are not that accessible in cyanobacteria (Laughlin et al., 2019). Another hypothesis suggests NdhS binds Fd^red^ with five positively charged lysine residues near its flexible C‐terminus, via a proposed ‘fly‐casting’ mechanism that bends Fd towards NdhI, where the first iron–sulfur cluster is located. This proposed binding mechanism is similar to Fd‐Fd‐NADP‐reductase interactions (Schuller et al., 2019). The potential presence of two Fd‐binding sites could enhance electron transport efficiency to plastoquinone (PQ) and minimize accumulation of the semi‐PQ radical intermediate during PQ reduction (Laughlin et al., 2019). Additionally, the loosely NDH‐1 associated subunit NdhV, that likely interacts with NdhS, may assist in Fd‐binding or accelerate the electron transfer from Fd^red^ to the electron transport chain, acting as a positive regulator of CET under high‐light stress (Zhang et al., 2020).

After binding of Fd^red^, a single electron is transferred onto an electron transport chain, which consists of three iron–sulfur ([4Fe‐4S]) clusters, namely N6a, N6b, and N2. N6a and N6b are coordinated by NdhI, while N2 is coordinated by the core subunit NdhK. Adjacent to the N2 cluster is the PQ‐binding site that is mainly formed by the OPS‐subunit NdhL. PQ‐binding is likely further stabilized by core subunits NdhA, NdhH, and NdhK. In a structure of NDH‐1MS from *T. elongatus*, it was shown that the hydrophobic PQ‐tail reaches into the membrane with the PQ‐binding site entrance stabilized by flanking lipids (Schuller et al., 2020). Structural OPS‐subunits NdhN and NdhM might be involved in binding elongated termini of the core subunits NdhH, NdhI, NdhJ, and NdhK in the peripheral arm, thereby covering hydrophobic patches of these termini, which would be surface‐exposed otherwise (Schuller et al., 2019). Besides the three identified iron–sulfur clusters, a fourth region with high electron density has been found in the peripheral arm of the NDH‐1L structure. This ‘X‐site’ is located at the interface of the subunits NdhA, NdhH, NdhK, and NdhM and has surface‐exposed positively charged and hydrophilic residues, potentially coordinating a yet unknown metal ‘X‐cofactor’. Furthermore, the X‐site seems to have a connection to the third iron–sulfur cluster N2 and could theoretically be another entry point for electrons into the electron transport and chain, preventing the accumulation of radical semi‐PQ in addition to the two Fd‐binding sites (Laughlin et al., 2019).

The peripheral and membrane arms of NDH‐1 complexes are connected via the subunits NdhA and NdhL. The membrane arm contains the small TM core subunits NdhC, NdhE, and NdhG and the antiporter‐like subunits NdhB, NdhD, and NdhF. The latter two are cyanobacteria‐specific, with four different isoforms of both proteins specific to one of the four NDH‐1 types with especially the NdhF isoforms fulfilling quite different functions (Schuller et al., 2020; Zhang et al., 2020). NdhD, together with the OPS‐subunits NdhP and NdhQ, connects these cyanobacterial subunits to the NDH‐1M common module. In NDH‐1L, the NdhF (NdhF1) subunit likely forms an additional proton channel, suggesting that NDH‐1 complexes seem to possess three to four proton channels. These channels are proposed to couple PQ reduction to the transport of protons over the membrane via a charge‐redistribution cascade already observed in the related respiratory complex I (Laughlin et al., 2019).

Structure and functions of the CO_2_‐uptake systems NDH‐1MS (NDH‐1_3_) and NDH‐1MS' (NDH‐1_4_) show significant differences compared to the other two NDH‐1 types, NDH‐1L and NDH‐1L'. While the latter use free energy released by the oxidoreduction between Fd and PQ only for proton pumping, NDH‐1MS and NDH‐1MS' additionally couple the oxidoreduction to the hydration of CO_2_ to form HCO_3_^−^ (Figure 3). This additional function is facilitated by the cyanobacteria‐specific module consisting of special isoforms of NdhD and NdhF, as well as the additional subunits CupA and CupS, which are absent in the structures of NDH‐1L and NDH‐1L'. CupA and CupS, short for CO_2_ uptake, form a second peripheral arm reaching into the cytoplasm at the distal side of the complex, leading to a U‐shaped architecture of the CO_2_‐uptake systems. In the structure of NDH‐1MS, CupA is connected to NdhF3 via electrostatic interactions. CupS binds to CupA and could be involved in stabilizing the interaction between CupA and NdhF3 (Schuller et al., 2020). The CA activity of NDH‐1MS occurs at the interface of CupA and NdhF3 forming a unique active site distinct from classical β‐type CAs. Remarkably, the CA activity of NDH‐1MS appears unidirectional, and hydrating CO_2_ to convert it into HCO_3_^−^, whereas classical CAs are typically bidirectional (Hagemann & Kaplan, 2020; Sun et al., 2019). Unlike the typical β‐sheet fold and His/Cys catalytic residues, CupA exhibits an α‐helical structure, with His130 and Arg135 coordinating a Zn^2+^ cofactor. Molecular dynamics modeling suggest that NdhF3 residues Tyr41, Glu114 and Arg37 facilitate catalysis. Arg37, activated via Zn^2+^, forms an ion pair with Glu114 and Tyr41, enabling Tyr41 to deprotonate an adjacent water molecule. The resulting hydroxide activates another Zn^2+^ coordinated H_2_O that performs a nucleophilic attack onto a CO_2_ molecule, forming a proton and HCO_3_^−^, the latter is released into the cytoplasm.

Modeling proposed a different rate‐limiting step in the mechanism and different energetics than usually seen in CAs to overcome the otherwise thermodynamically unfavorable CO_2_‐to‐HCO_3_^−^ conversion in the cytoplasm (Hagemann & Kaplan, 2020; Schuller et al., 2020). This is likely achieved by coupling the hydration reaction to proton transfer between CupA and NdhD3. The proton generated in the CO_2_ hydration is removed through protonic connection between subunits CupA and NdhD3 to protonate a loading site in the proton channel of NdhD3 and subsequently transported into the thylakoid lumen. The driving force of this proton removal is the oxidoreduction in the peripheral arm, where PQ reduction and PQH_2_ release trigger a charge‐redistribution cascade, which coordinates proton translocation and opening of the CA site. The free energy from this exergonic oxidoreduction is calculated to be sufficient for compensating the energies required for both endergonic proton pumping and CO_2_‐hydration (Schuller et al., 2020).

Interestingly, a hydrophobic channel was identified in the NdhF3 subunit of NDH‐1MS connecting the CA active site to the thylakoid lumen. This channel likely serves as an entry for CO_2_ into the active site, proposing that NDH‐1MS actually hydrates CO_2_ originating from the thylakoid lumen rather than the cytoplasm. Structural analyses further support this idea, as the active site is shielded by hydrophobic residues on the cytoplasmic side, preventing CO_2_ access from that direction (Schuller et al., 2020). This finding contradicts the previous theory of the cyanobacterial CCM function, in which cytoplasmic CO_2_ was mainly proposed to arise from passive diffusion of CO_2_ into the cell, carboxysome leakage, and pH‐dependent conversion of HCO_3_^−^ to CO_2_. Instead, the new findings suggest HCO_3_^−^ leakage into the acidic thylakoid lumen generates CO_2_ which must be rehydrated to HCO_3_^−^ by the CO_2_‐uptake systems (Hagemann & Kaplan, 2020). However, this hypothesis is solely based on structural data; additional biochemical evidence is required to confirm the proposed function of the CO_2_‐channel.

## REGULATION OF THE CYANOBACTERIAL CCM

Given the dynamic nature of their habitats, where light intensity and CO_2_ availability fluctuate constantly, efficient control of photosynthetic performance and Ci assimilation is essential for sustaining cyanobacterial growth. In response to environmental shifts, particularly under inorganic carbon limiting conditions, cyanobacteria employ a sophisticated regulatory network that fine‐tunes the CCM through signaling cascades involving transcriptional regulators (TRs), protein phosphorylation, and allosteric enzyme regulators. Integral metabolic signals such as 2‐oxoglutarate (2‐OG), 2‐PG, and cyclic AMP (cAMP) modulate the abundance of RubisCO, Ci transporters, and carboxysome components to adapt their abundance to cellular requirements (Bolay et al., 2022; Jiang et al., 2018; Selim et al., 2018; Takahashi et al., 2004; Wang et al., 2004).

### Transcriptional regulation of CCM genes

**Figure 4:**
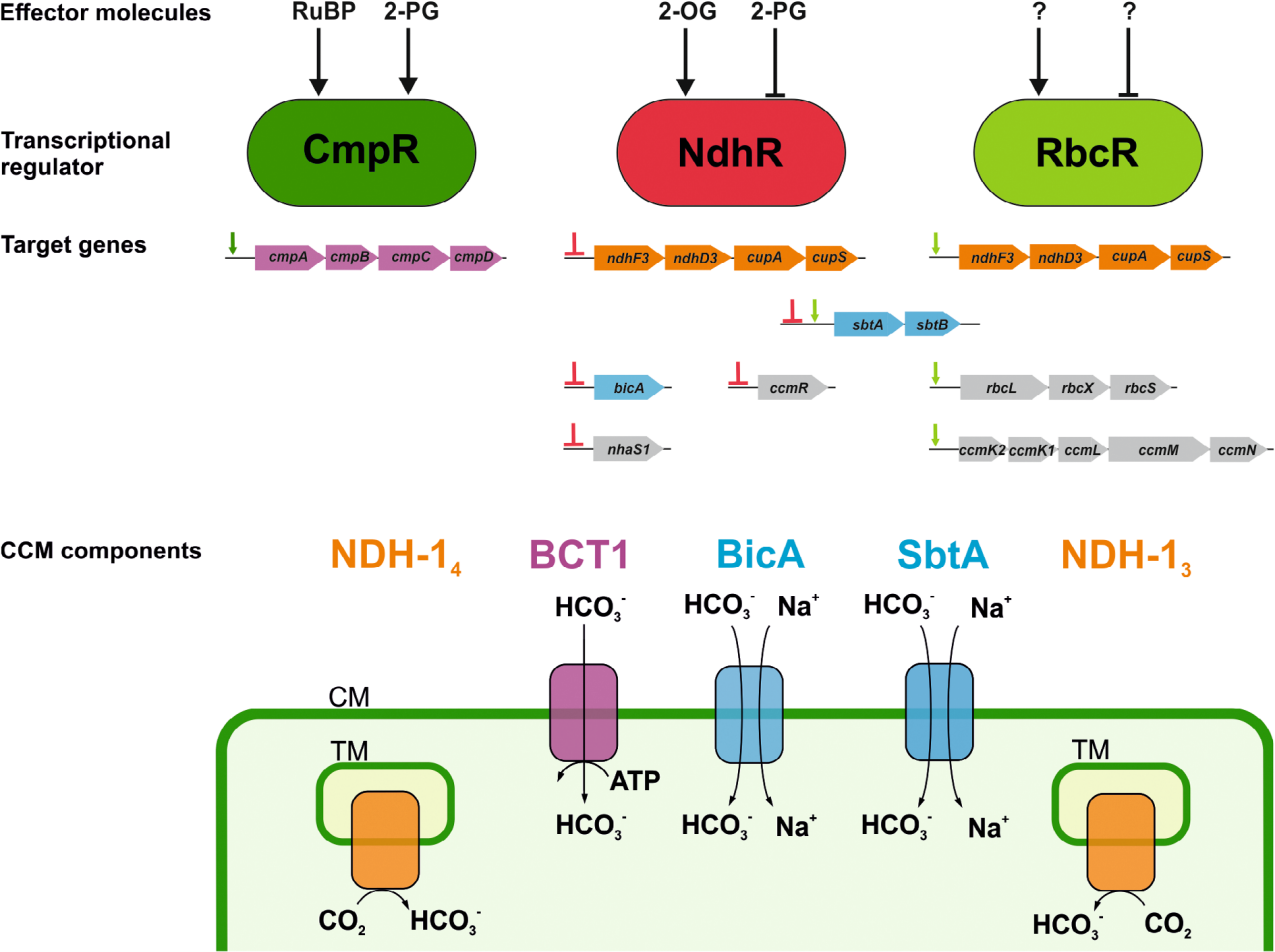
Key transcriptional regulators of the carbon‐concentrating mechanism (CCM). The transcriptional activators CmpR and RbcR, together with the transcriptional repressor NdhR, ensure precise regulation of CCM gene expression. Under conditions of low inorganic carbon (Ci) availability, CmpR primarily activates the *cmp* operon, whereas RbcR acts as a master activator of multiple CCM‐associated genes, including those encoding high‐affinity Ci transporters, RubisCO subunits, and carboxysome shell proteins. In contrast, under high‐Ci conditions, expression of CCM components is repressed by NdhR. DNA affinity of the regulators is modulated by specific effector metabolites, such as ribulose‐1,5‐bisphosphate (RuBP), 2‐phosphoglycolate (2‐PG), or 2‐oxoglutarate (2‐OG), that accumulate under defined inorganic carbon availability, thereby coupling intracellular metabolic status to CCM gene expression.

TRs play central roles in coordinating cellular responses in cyanobacteria, including those triggered by fluctuating Ci availability and light conditions (Figure 4). A shift from HC (high Ci, >1% CO_2_ [v/v]) to LC (low Ci, 0.04% CO_2_ [v/v]) conditions triggers extensive cellular reprogramming, including upregulation of genes encoding numerous CCM components (Burnap et al., 2015). Full acclimation to LC conditions additionally requires activation of genes beyond the CCM, including the operon encoding flavodiiron proteins Flv2 and Flv4, which protect photosystem II from photooxidative stress, as well as other stress‐response genes and nitrogen assimilation genes that adjust the cellular carbon/nitrogen balance (Wilde & Hihara, 2016). In addition, sigma factor C (SigC) was found to regulate Ci levels by formation of an RNA polymerase‐SigC holoenzyme that controls the expression of photosynthetic and CCM genes in response to light and Ci signals (Kurkela et al., 2025). Mechanistically, these transcriptional changes are mediated by positive and negative TRs that can sense the cell's Ci status through metabolic signals. Transcriptional activators bind to specific DNA motifs located near transcriptional start sites (TSSs) and thereby facilitate RNA polymerase recruitment and enhance transcription. Conversely, transcriptional repressors occupy promoter regions to block RNA polymerase binding, thereby inhibiting gene expression. To date, five TRs have been associated with the transcriptional control of CCM genes in cyanobacteria: NdhR, CmpR, RbcR, cyAbrB2 and SyCRP1. Nevertheless, a recent study analyzing a low CO_2_‐responsive gene co‐expression network in *Synechocystis* identified novel TRs, encoded by *slr6040* and *sll5097*, that could facilitate the photosynthetic growth of *Synechocystis* under low Ci conditions. However, the relevance of these findings requires further investigation (Wu et al., 2026).

#### NdhR

The NAD(P)H dehydrogenase regulator (NdhR) belongs to the LysR‐type TR (LTTR) family, a widely conserved group of prokaryotic regulators that undergo effector‐dependent conformational changes that modulate their DNA‐binding properties (Schell, 1993). In *Synechocystis*, NdhR is encoded by the gene *ccmR* (*sll1594*) and was initially identified as a regulator of *ndh* genes, which gave rise to its original designation (Figge et al., 2001). NdhR functions predominantly as a transcriptional repressor of multiple CCM‐associated genes under HC conditions (Figure 4), with repression relieved upon a shift to LC conditions (Figge et al., 2001; Wang et al., 2004; Woodger et al., 2007). Key targets include the *ndh*‐1_3_ operon (*ndhF3/ndhD3/cupA/sll1735*) encoding components of the specialized high‐CO_2_‐affinity NDH‐1 complexes, the sodium‐dependent bicarbonate transporter encoded by *sbtA/B* and *bicA*, the Na^+^/H^+^ antiporter *nhaS1*, *ccmR* itself, and several noncoding RNAs (Klähn et al., 2015; Wang et al., 2004). Given its central regulatory role in the CCM, the new name CcmR (Ci‐concentrating mechanism regulator) was proposed and is for instance used in other cyanobacteria such as *Synechococcus* sp. PCC 7002 (McClure et al., 2016; Woodger et al., 2007). The consensus NdhR‐binding motif ATAG‐N_8_‐CTAT is typically located upstream of the first gene of each regulated operon (Klähn et al., 2015).

NdhR activity is modulated by small‐molecule effectors that reflect the metabolic state of the cell. The TCA‐cycle intermediate 2‐OG, the carbon skeleton that accepts assimilated nitrogen, accumulates upon high Ci or nitrogen limitation (Forchhammer & Selim, 2020). Binding of 2‐OG to NdhR leads to increased affinity of the TR to its binding sites in promoters of target genes and subsequently, their expression is repressed (Daley et al., 2012). Under prolonged high‐light and LC conditions, RubisCO becomes CO_2_‐limited, leading to elevated synthesis of 2‐PG via the oxygenase reaction of the enzyme. Simultaneously, 2‐OG concentrations drop because diminished carbon assimilation leads to an N‐oversupply relative to carbon flux through the GS‐GOGAT cycle. Consequently, NdhR mostly occurs in the 2‐PG‐bound state, in which the affinity of the repressor to its DNA‐binding sites is reduced, resulting in derepression of target genes (Daley et al., 2012; Jiang et al., 2018).

The structural basis of this effector‐dependent regulation was elucidated by Jiang et al., who solved the crystal structure of NdhR as a tetrameric LTTR composed of two compact and two extended subunits (Jiang et al., 2018). Each subunit contains a DNA‐binding domain (DBD), featuring a winged helix‐turn‐helix motif formed by four α‐helices (α1–α4) and two β‐strands (β1–β2), and a regulatory domain (RD) composed of two α/β Rossmann‐like subdomains connected by crossover β‐sheets. The tetramer forms a dimer‐of‐dimers, where dimers consist of one compact and one extended monomer linked via their α4 helices, and two such dimers associate via interactions between their RDs. Structural analyses showed that 2‐OG binds at the interface of two RDs, interacting with residues Ser224, Asn225, Tyr105, and Lys10, and the corresponding residues of the opposite RD, thereby stabilizing the NdhR‐2‐OG complex and promoting high‐affinity DNA‐binding. In contrast, 2‐PG binds within the cleft between RD1 and RD2, forming hydrogen bonds through its phosphate group with Arg195, His130, Thr201, and Thr101, and via its carboxyl group with Thr102, Asn164, and Arg267. Binding of 2‐PG induces conformational rearrangements throughout the tetramer that weaken DNA binding. Through this effector‐dependent regulation, NdhR coordinates expression of CCM components in response to the fluctuating carbon/nitrogen ratio.

#### CmpR

CmpR is a second LTTR, that, in contrast to NdhR, functions predominantly as a transcriptional activator (Nishimura et al., 2008; Takahashi et al., 2004) (Figure 4). Unlike the globally acting NdhR, CmpR regulates a more restricted set of targets. It activates the transcription of the *cmpABCD* operon, encoding the ABC‐type bicarbonate transporter BCT1, under LC and/or high‐light conditions (Omata et al., 2001; Takahashi et al., 2004). In *Synechocystis*, this regulatory role is further highlighted by the genomic context: *cmpR* (*sll0030*) is located immediately upstream of the *cmp* operon (*slr0040*‐*0044*) but is transcribed divergently (Omata et al., 2001), a genomic arrangement characteristic of many LTTRs (Schell, 1993). Beyond its role in *cmp* activation, CmpR also elevates LC‐ and high‐light‐induced gene expression of *psbAII* and *psbAIII* (Takahashi et al., 2004), encoding the D1 protein of the photosystem II reaction center, by binding to enhancer elements located downstream of their respective transcriptional start site (TSS). CmpR has further been shown to autoregulate expression of its own gene under HC conditions in *S. elongatus* (Pan et al., 2016). Its consensus DNA‐binding motif, TTA‐N_7_/_8_‐TAA, provides a sequence‐specific basis for target promotor recognition (Tanaka et al., 2008).

Regulatory activity of CmpR is stimulated by the co‐inducers 2‐PG and RuBP, which enhance DNA‐binding affinity (Nishimura et al., 2008; Takahashi et al., 2004). RuBP accumulates under LC due to reduced flux through the CBB cycle. Mahounga et al. resolved the crystal structure of the CmpR RD in complex with RuBP, providing key insights into the effector‐mediated activation mechanism (Mahounga et al., 2018). As in other LTTR, each RD comprises two α/β Rossmann‐like subdomains, each formed by a five‐stranded β‐sheet flanked by three α‐helices. The two subdomains are connected through a characteristic crossover of antiparallel β‐strands (β4 and β10), a structural feature consistent with other LysR‐type TRs. RuBP binds at the positively charged interface formed between two RD monomers. One phosphate group of RuBP is coordinated by residues Lys106, Ser226, and Asn227 of RD1 along with Lys106′ from the partnering RD, while the second phosphate group is stabilized by the equivalent residues of the opposing subunits (Mahounga et al., 2018). Through this effector‐dependent mechanism, CmpR serves as an important activator of bicarbonate uptake during Ci limitation and high‐light stress.

#### RbcR

RbcR, encoded by *rbcR* (*sll0998*) and alternatively termed CBB cycle regulator (CbbR), is another member of the LTTR family. It functions primarily as a transcriptional activator under LC conditions, inducing expression of RubisCO genes *rbcLXS* as well as multiple key CCM components, including *sbtA*, the *ndhF3* operon, and the *ccmK2K1LMN* operon (Bolay et al., 2022) (Figure 4). RbcR also positively regulates its own expression during LC stress. The RbcR regulon likely extends beyond the canonical CCM and includes additional loci, such as the *flv4‐sll0218‐flv2* operon encoding flavodiiron proteins Flv2 and Flv4. The consensus DNA‐binding motif, ATTA(G/A)‐N_5_‐(C/T)TAAT, mediates activation when located approximately 90 nucleotides upstream of the TSS but can also repress gene expression when positioned ~10 nucleotides upstream of the TSS, highlighting the versatility of its regulatory interactions (Bolay et al., 2022). RbcR activity is modulated by small effector molecules RuBP, 3‐PGA, and NADPH in the glaucophyte *Cyanidioschyzon merolae* (Minoda et al., 2010), and by phosphoenolpyruvate in *Cupriavidus necator* N16 (Gruber et al., 2017), however, little is known about cyanobacterial effector molecules which yet have to be identified. Post‐transcriptional control of RbcR has been found to occur via interaction with the site‐2 protease Sll0528 (Lin et al., 2025). Although no crystal structure of a cyanobacterial RbcR has been resolved to date, its classification as a LTTR suggests that its overall architecture likely resembles that of structurally characterized LTTR family members.

#### cyAbrB2 and SyCRP1

Beyond the major LysR‐type TRs, cyanobacterial CCM regulation involves additional regulators, including SyCRP and cyAbrB2. Cyanobacteria harbor a conserved family of AbrB‐like TRs, with at least one homolog present in every sequenced cyanobacterial genome (Sakr et al., 2013). *Synechocystis* sp. PCC 6803 contains two members of this family, cyAbrB1 (*sll0359*) and cyAbrB2 (*sll0822*). CyAbrB1 appears to be essential for cell viability, as repeated attempts to generate knockout mutants have failed (Ishii & Hihara, 2008; Oliveira & Lindblad, 2009). In contrast, Δ*cyabrB2* mutants have successfully been constructed, indicating a non‐essential but potentially regulatory role (Ishii & Hihara, 2008). cyAbrB2 has been shown to support the regulatory activities of NdhR and CmpR and as a transcriptional activator for a subset of CCM genes, including the *ndh*‐1_3_ and *cmp* operons (Orf et al., 2016). An earlier study also reported that cyAbrB2 represses *sbtA/B* expression (Lieman‐Hurwitz et al., 2009); however, this finding was not reproduced in a subsequent analysis (Orf et al., 2016). Beyond its involvement in CCM regulation, cyAbrB2 influences additional global regulatory networks, including the NtcA (global nitrogen transcription regulator; Ishii & Hihara, 2008) and LexA (global regulator, for example for bidirectional hydrogenase Hox; Kariyazono & Osanai, 2024) regulons, phycobilisome subunit genes (*apcE, apcF, cpcG2*), and components of photosystems I and II (*psbK, psbM, psbZ, psbO, psbV*). Notably, Δ*cyabrB2* mutants fail to induce *ndhR* expression under LC conditions, suggesting that CyAbrB2 may also activate expression of the key CCM repressor (Orf et al., 2016).

Two cAMP receptor proteins (CRPs) have been identified in *Synechocystis*: SyCRP1 (*sll1371*) and SyCRP2 (*sll1924*) (Yoshimura et al., 2000). Both proteins form a complex required for twitching motility (Song et al., 2018; Yoshimura, Yoshihara, et al., 2002). Moreover, both SyCRP1 and SyCRP2 were found to form a complex with ComFB, a new c‐di‐NMP receptor protein, thereby modulating the DNA‐binding activity or stability of these transcription factors in response to c‐di‐GMP signaling (Samir, Doello, et al., 2025; Samir, Elshereef, et al., 2025; Wallner et al., 2026). In addition, SyCRP1 functions as a TR for a subset of CCM genes (Yoshimura, Yanagisawa, et al., 2002). Under LC conditions, SyCRP1 is predominantly membrane‐associated, but is released into the cytosol upon binding cAMP under HC conditions (Bantu et al., 2022). This mechanism enables SyCRP to act as an indirect sensor of intracellular CO_2_ levels. Elevated CO_2_ activates adenylate cyclase activity, increasing intracellular cAMP concentrations (Hammer et al., 2006). cAMP binding to SyCRP triggers its release from the membrane, allowing the protein to bind regulatory regions upstream of CCM genes and modulate their transcription (Bantu et al., 2022; Chauhan et al., 2025). Transcriptomic analyses of a SyCRP1 knockout strain revealed upregulation of multiple CCM‐associated genes under HC conditions, including the *cmp*, *ndh‐1*_*3*_, and *sbtA/B* operons, multiple genes encoding NDH‐1 complex components, *ndhR*, and the shell protein genes *ccmK1*, *ccmK2*, and *ccmK3*, indicating that SyCRP1 functions as a co‐repressor of these targets (Bantu et al., 2022, Chauhan et al., 2025). Direct SyCRP1 binding to promoter regions upstream of sbtA and ccmK3 has been experimentally verified, underscoring its importance as an additional regulatory layer within the CCM network (Bantu et al., 2022). Despite these advances, further studies are required to elucidate the structural basis of SyCRP1 function, including its interactions with cAMP and DNA.

As illustrated by these regulatory mechanisms, transcriptional control of CCM genes results from the coordinated action of multiple regulators, allowing precise tuning of expression in response to changing environmental conditions. For example, *sbtA* and *sbtB* are activated under LC by RbcR and cyAbrB2, whereas their transcription is repressed under HC by NdhR (Bolay et al., 2022; Wang et al., 2004). In addition to transcriptional regulation, the CCM is modulated by layers of post‐transcriptional and post‐translational control, including asRNA‐mediated regulation, protein phosphorylation, and allosteric modulation of key enzymes.

### Post‐ transcriptional and translational regulation

In addition to TRs that modulate transcript abundance, several post‐transcriptional regulatory mechanisms adjust protein levels and activity to rapid changes in Ci abundance and light availability. Numerous noncoding RNAs (ncRNAs), that accumulate under LC conditions, are involved in that regulatory network to potentially provide additional layers of transcriptional fine‐tuning and translational control (Eisenhut et al., 2012; Klähn et al., 2015). Protein phosphorylation also acts as a rapid, post‐translational switch that assists in fine‐tuning the activity of Ci transporters. The bicarbonate transporters SbtA, BicA, and BCT1 are believed to be inactive in darkness to avoid futile ion cycling and are rapidly reactivated in the light under Ci limitation, through post‐translational phosphorylation (Price, 2011; Price et al., 2008). This mechanism ensures efficient balancing of energy use with Ci acquisition.

The sodium‐dependent bicarbonate transporter SbtA has an additional level of allosteric regulation mediated by the small PII‐like protein SbtB (Selim & Alva, 2024). SbtB functions as a sensor of cellular energy, carbon, and redox status (Selim et al., 2018, 2023). It is a highly conserved homotrimeric protein with a ferredoxin‐like fold (Kaczmarski et al., 2019; Selim et al., 2018), characteristic of the PII superfamily (Forchhammer et al., 2022). Like other PII proteins, SbtB contains multiple regulatory loop structures (namely B‐, C‐, T‐, and R‐loop) (Liu et al., 2021; Selim et al., 2020; Selim & Alva, 2024). The flexible T‐loop mediates the interaction with SbtA and undergoes effector‐dependent conformational changes. B‐ and C‐loops are positioned near the inter‐subunit clefts and bind effector molecules (Forchhammer & Selim, 2020), including ATP, ADP, AMP, and the second messengers cAMP and c‐di‐AMP (Selim et al., 2018, 2023). AMP accumulation signals Ci limitation, cAMP reflects HC conditions, ATP and ADP report the cellular energy state, and c‐di‐AMP shows diurnal oscillations with a daytime peak (Mantovani et al., 2023; Selim & Alva, 2024). In *Synechocystis*, the presence of elevated AMP under Ci limitation promotes SbtA‐SbtB complex formation, whereas cAMP accumulation under Ci‐replete conditions induces complex dissociation (Selim et al., 2018). Interestingly, it was observed that the energetically costly bicarbonate pool leaked out of *Synechocystis* cells on relatively short time‐scales, whereas the Δ*sbtB* mutant showed even higher leakage (Haffner et al., 2023). Recent structural studies revealed the mechanism of SbtB‐dependent regulation of SbtA, in which SbtB seems to act as a valve plug to control the transport activity of SbtA and prevent bicarbonate leakage (Fang et al., 2021; Haffner et al., 2023; Selim & Alva, 2024). However, another study in which SbtA and SbtB were co‐expressed in *E. coli* suggested an inhibitory effect of SbtB on SbtA (Du et al., 2014). Nevertheless, this observation may not be directly related or comparable to cyanobacteria, as *E. coli* lacks a CCM; consequently, SbtA/SbtB expression and regulation in this heterologous system are not subject to the LC‐dependent control found in cyanobacteria. In cyanobacteria, the interaction between SbtB and SbtA appears to occur under LC conditions, when increased HCO_3_^−^ uptake is required (Selim et al., 2018). Therefore, the current model based on several structural analyses proposes that SbtB may not inhibit transport but instead inactivates SbtA and prevents the leakage of the energetically expensive HCO_3_^−^ only in darkness due to the absence of photosynthesis (Haffner et al., 2023). Nevertheless, there appear to be some differences in interpretation in the literature relating to SbtB function, with some suggestions that SbtB inhibits the HCO_3_^−^ transport activity of SbtA (Förster et al., 2023). Despite this, it is evident that SbtB primarily acts as a blocking mechanism that operates in response to adenylate energy sensing to prevent HCO_3_^−^ pumping or leakage—a function that remains questionable yet—when internal Ci pool sizes have achieved a pseudo‐steady‐state maximum (Förster et al., 2023; Haffner et al., 2023; Selim et al., 2023; Selim & Alva, 2024). Recent findings further indicate that an additional player, the small protein SbtC, may be required for efficient SbtA‐mediated bicarbonate transport (Walke et al., 2026). Interestingly, the newly discovered SbtA2 protein is coupled to another PII‐like protein that we termed SbtB2 or SbtB‐like (Rourke et al., 2026a; Selim & Alva, 2024). However, the mechanism by which SbtB2 regulates SbtA2 remains to be elucidated.

Beyond ligand‐mediated control, SbtB integrates redox signals through its conserved R‐loop/C‐terminal hairpin containing the CGPxGC motif, whose cysteines form a disulfide bridge under oxidizing conditions (Mantovani et al., 2024; Selim et al., 2023). These redox‐dependent conformational changes modulate apyrase activity of SbtB. In the reduced state, the T‐loop adopts an ATP‐protective conformation, whereas in the oxidized state ATP can be hydrolyzed to ADP and subsequently AMP (Selim et al., 2023). This mechanism supports the hypothesis that the oxidized state at nighttime drives SbtB toward an AMP‐bound state, promoting its association with SbtA and contributing to closure of the SbtA transport channel at night, although the precise molecular mechanism remains to be elucidated. Also, it seems that SbtB integrates additional red/far‐red light signals to regulate the activity of CCM by yet an unknown mechanism (Oren et al., 2021).

In addition to regulating SbtA, SbtB has been found to modulate additional cellular processes such as the c‐di‐AMP‐dependent activation of the glycogen branching enzyme GlgB during the day (Haffner et al., 2025; Selim et al., 2021). Moreover, it was found that SbtB interacts physically with both BicA and the ATPase subunits of the BCT1 complex (CmpC/D) (Haffner et al., 2025). However, the molecular details of these interactions and the influence of SbtB on BCT1 and BicA activities require further investigation. Interestingly, similar to SbtB, classical PII was proposed to regulate nitrate uptake through the interaction with the ABC‐type nitrate transporter NrtABCD (CmpABCD paralog), in which the ATPase subunit NrtC is fused to a CRD (Forchhammer et al., 2022; Li et al., 2024). Structural analysis revealed that the core structure of PII buries the CRD of NrtC, thereby narrowing the NrtB substrate translocation channel and locking the transporter in an inhibited inward conformation (Li et al., 2024). Alphafold structural prediction revealed a similar mechanism for SbtB on the CmpC subunit of the BCT1 complex (Figure 3). SbtB further influences transcriptional responses: deletion of SbtB induces an LC‐pre‐acclimated state and derepresses numerous genes under HC conditions (Mantovani et al., 2022). Although SbtB lacks a DBD, these effects may be mediated indirectly through interaction with TRs via an unidentified mediator protein. These observations reflect a broader impact of SbtB on CCM in particular and reinforce the versatility of SbtB in controlling carbon homeostasis. Collectively, these transcriptional and post‐transcriptional mechanisms operate coordinatively to ensure coordinated regulation of the CCM and broader cellular metabolism under fluctuating Ci conditions.

## CONCLUSION AND PERSPECTIVES

Fundamental achievements have been made in understanding the function, regulation and structural assembly of the cyanobacterial CCM and its components, which now provides a foundation for a more integrated view of CCM biogenesis and control. These insights not only deepen our understanding of microbial carbon metabolism but also open promising avenues for synthetic biology and practical engineering. Key CCM modules can be heterologously expressed, correctly assembled and function outside their native hosts, for example in engineered *E. coli*, showing that coordinated expression of transporters, CA and shell components can create a high‐CO_2_ microenvironment for RubisCO (Flamholz et al., 2020). For instance, a recent study established a synthetic CO_2_‐recycling platform in *E. coli* by integrating cyanobacterial carbonic anhydrases together with RubisCO and phosphoribulokinase to drive carbon recycling and 5‐aminolevulinic acid biosynthesis, a valuable prodrug (Effendi & Ng, 2026). This also creates a lot of opportunities to improve the photosynthetic performance of crop plants by introducing CCM components to either pump HCO_3_^−^ near RubisCO or compartmentalize RubisCO within synthetic carboxysomes (Chen, Hojka, et al., 2023; Lin, Occhialini, Andralojc, Devonshire, et al., 2014; Lin, Occhialini, Andralojc, Parry, & Hanson, 2014; Long et al., 2018; Ni et al., 2022; Pengelly et al., 2014; Rottet et al., 2021, 2024; Rourke et al., 2026a). For example, some advances have been achieved in engineering α‐carboxysomes into plant chloroplasts to support autotrophic photosynthesis (Chen, Hojka, et al., 2023). Additionally, the overexpression of β‐carboxysomes increased the photosynthesis and growth of *Synechocystis* (Hu et al., 2026). Beyond improving plant CO_2_ fixation, carboxysomes are attractive for biotechnological applications: their self‐assembling, modular shells can be repurposed for engineering minimal organelles or microcompartments to concentrate substrates, segregate pathways, and enhance both natural and novel metabolic reactions.

## CONFLICT OF INTEREST

The authors declare no conflict of interest.

## Data Availability

No data was used for the research described in the article.
